# Dynamic transcriptomes of resistant and susceptible peach lines after infestation by green peach aphids (*Myzus persicae* Sülzer) reveal defence responses controlled by the *Rm3* locus

**DOI:** 10.1186/s12864-018-5215-7

**Published:** 2018-11-28

**Authors:** Liang Niu, Lei Pan, Wenfang Zeng, Zhenhua Lu, Guochao Cui, Meili Fan, Qiang Xu, Zhiqiang Wang, Guohuai Li

**Affiliations:** 1 Key Laboratory of Horticultural Plant Biology, Ministry of Education, Huazhong Agricultural University, Wuhan, 430070 China; 2 Key Laboratory of Fruit Breeding Technology of Ministry of Agriculture, Zhengzhou Fruit Research Institute, Chinese Academy of Agricultural Sciences, Zhengzhou, 450009 China

**Keywords:** *Rm3* locus, Peach, *Myzus persicae* (Sülzer), Innate immunity, Transcriptome analysis, Aphid resistance

## Abstract

**Background:**

The green peach aphid (GPA), *Myzus persicae* (Sülzer), is a widespread phloem-feeding insect that significantly influences the yield and visual quality of peach [*Prunus persica* (L.) Batsch]. Single dominant gene (*Rm3*)-based resistance provides effective management of this invasive pest, although little is known about the molecular responses of plants to GPA feeding.

**Results:**

To illustrate the molecular mechanisms of monogenic resistance in peach to young tissue-infecting GPAs, aphid-resistant/aphid-susceptible peach lines from a segregating population with *Rm3/rm3* and *rm3/rm3* genotypes were infested with GPAs for 3 to 72 h. Transcriptome analysis of the infested tissues identified 3854 differentially expressed genes (DEGs). Although the majority of the DEGs in the resistant line also responded to aphid attack in the susceptible line, the overall magnitude of change was greater in the resistant line than in the susceptible line. The enriched gene ontology of the 3854 DEGs involved in plant defence responses included redox situation, calcium-mediated signalling, transcription factor (e.g., WRKY, MYB, and ERF), MAPK signalling cascade, phytohormone signalling, pathogenesis-related protein, and secondary metabolite terms. Of the 53 genes annotated in a 460 kb interval of the *rm3* locus, seven genes were differentially expressed between the aphid-resistant and aphid-susceptible peach lines following aphid infestation.

**Conclusions:**

Together, these results suggest that the *Rm3*-dependent resistance relies mainly on the inducible expression of defence-related pathways and signalling elements within hours after the initiation of aphid feeding and that the production of specific secondary metabolites from phenylpropanoid/flavonoid pathways can have major effects on peach-aphid interactions.

**Electronic supplementary material:**

The online version of this article (10.1186/s12864-018-5215-7) contains supplementary material, which is available to authorized users.

## Background

The green peach aphid (GPA) (*Myzus persicae* Sülzer), one of the most generalist aphid species, is capable of feeding on a wide range of food and ornamental crops from over 40 plant families in temperate regions and commonly uses peach, *Prunus persica* (L.) Batsch, as a primary host in spring [1]. The GPA ingests phloem sap from the host plant’s sieve elements through narrow mouthparts called stylets [2]. Aphid feeding causes minimal damage to the hosts compared with the feeding of chewing insects. However, aphid infestation causes heavy damage to trees due to the penetration of the leaves by the stylet, which is responsible for leaf curling, heavy breakdowns in shoot growth, reduced fruit quality and fruit malformation [3]. GPA infestation causes further harm by spreading viruses, including the plum pox potyvirus (PPV), which is the world’s most serious *Prunus* species disease [4]. Spraying insecticides is the primary method of controlling GPAs in peach orchards, although spraying results in increased production costs and environmental problems. Continuous use of insecticides has led to resistance to most classes of insecticides [5]. In addition, beneficial insects can be negatively affected by the continual usage of chemical insecticides [6].

To overcome the threat of GPAs, it is important to understand the peach immune system. Two layers of plant immunity are well defined: pattern-triggered immunity (PTI) and effector-triggered immunity (ETI) [7]. As the first line of the innate immune response, PTI is triggered by the perception of herbivore-associated molecular patterns (HAMPs) in the case of herbivory and microbe-associated molecular patterns (MAMPs) or pathogen-associated molecular patterns (PAMPs) in the case of infection by cognate plasma membrane-localized pattern recognition receptors (PRRs) [8]. Examples of HAMPs detected by plants include nematode pheromones (ascarosides), which are conserved among nematodes [9], and other components found in insect oral secretions (proteins, fatty acid-amino acid conjugates, sulphur-containing fatty acids) [10]. Plants recognize MAMPs/HAMPs and then initiate PTI with various defence responses, including the production of reactive oxygen species (ROS), deposition of callose, and reprogramming of the transcriptome to activate defences. [11]. To overcome this first line of defence pathways, adapted pathogens or herbivores can release may types of effectors that inhibit PTI into plant cells [7]. ETI, another pathogen-sensing mechanism mediated by plant resistance (R) proteins, can activate strong immune responses against pathogens and pests [12]. Generally, ETI is associated with strong immune responses, typically including the programmed cell death of plant cells at infection sites, which is called a hypersensitive response (HR) [13].

Host plant resistance seems to be the most favourable pest management method for environmental, economic, and social reasons [14]. In the past few years, five loci conferring resistance to phloem-feeding insects have been cloned. These loci include the tomato *Mi-1.2* gene [15] and the melon (*Cucumis melo*) *Vat* gene, which confer resistance to the aphids *Macrosiphum euphorbiae* and *Aphis gossypii*, respectively [16], and the rice genes *Bph3*, *Bph14*, and *Bph26*, which confer resistance to brown planthopper (*Nilaparvata lugens*) [17]. Five GPA-resistant genotypes from peach were identified in previous studies from the Institut National de la Recherche Agronomique, France (INRA); of these genotypes, two peach cultivars, “Weeping Flower Peach” and “Rubira”, were controlled by a single dominant gene, i.e., *Rm1* and *Rm2* (Rm for resistance to *M. persicae*), respectively [18–20]. Both genotypes showed a strong antixenosis-type resistance that prevented plant colonization by aphids; most GPAs left the plants within the first week following infestation, and approximately half left within the first 2 days [18]. In addition, reddish hypersensitive-like necrotic spots appeared at the puncture point on the apices of both genotypes within 2–3 days after the infestation [18]. A third antixenosis-type resistance genotype, which was also controlled by the single dominant gene *Rm3*, was identified in “Fen Shouxing” (*P. persica* var. densa Makino) by our recent study [21]. Interestingly, although induced systemic resistance was only demonstrated for *Rm2*-type resistance [19, 20], *Rm1*, *Rm2* and *Rm3* were mapped to the same narrow region (approximately 3 Mb) in chromosome 1 of the peach genome [22–24], and whether the GPA resistances conferred by “Weeping Flower Peach”, “Rubira” and “Fen Shouxing” are controlled by one distinct locus is still a question.

Plant defences against phloem-feeding insects involve multiple signalling cascades and metabolic pathways, and molecular genetic studies on the model plant *Arabidopsis thaliana* have demonstrated that LRR family receptor-like kinases, calcium signalling proteins, hormone synthesis and signalling, reductant/oxidant (redox) signalling, transcription factors, and secondary metabolic pathways are at least partially activated by phloem feeding [25]. The influences of phloem-feeding insects, such as aphids, on plant transcriptomes have been well studied in susceptible and resistant plants in many species, including *Arabidopsis*, tomato, soybean, grape, rice, wheat, and maize [25]. However, there is no information about the molecular mechanism of peach resistance to GPAs.

Foyer et al. [25] reviewed previous transcriptome profiling studies on plants exposed to phloem-feeding insect attack and reported that there is a lack of understanding of the dynamic interactions between hosts and aphids, especially for fruit trees such as peach. The present study aimed to investigate the global responses of peach to GPA infestation and identify the genes associated with these resistance responses by comparing the differential gene expression of *Rm3*-type resistant (*Rm3*/*rm3*) and susceptible (*rm3*/*rm3*) peach lines with RNA sequencing-based gene expression profiling analysis. The results of this study provide insights into the interactions between peach and *M. persicae* and identify transcriptomic changes in peach that may be associated with resistance to *Myzus persicae*.

## Results

### Dynamic analysis of resistance phenotypes against GPAs in R36 and S38

**Fig. 1:**
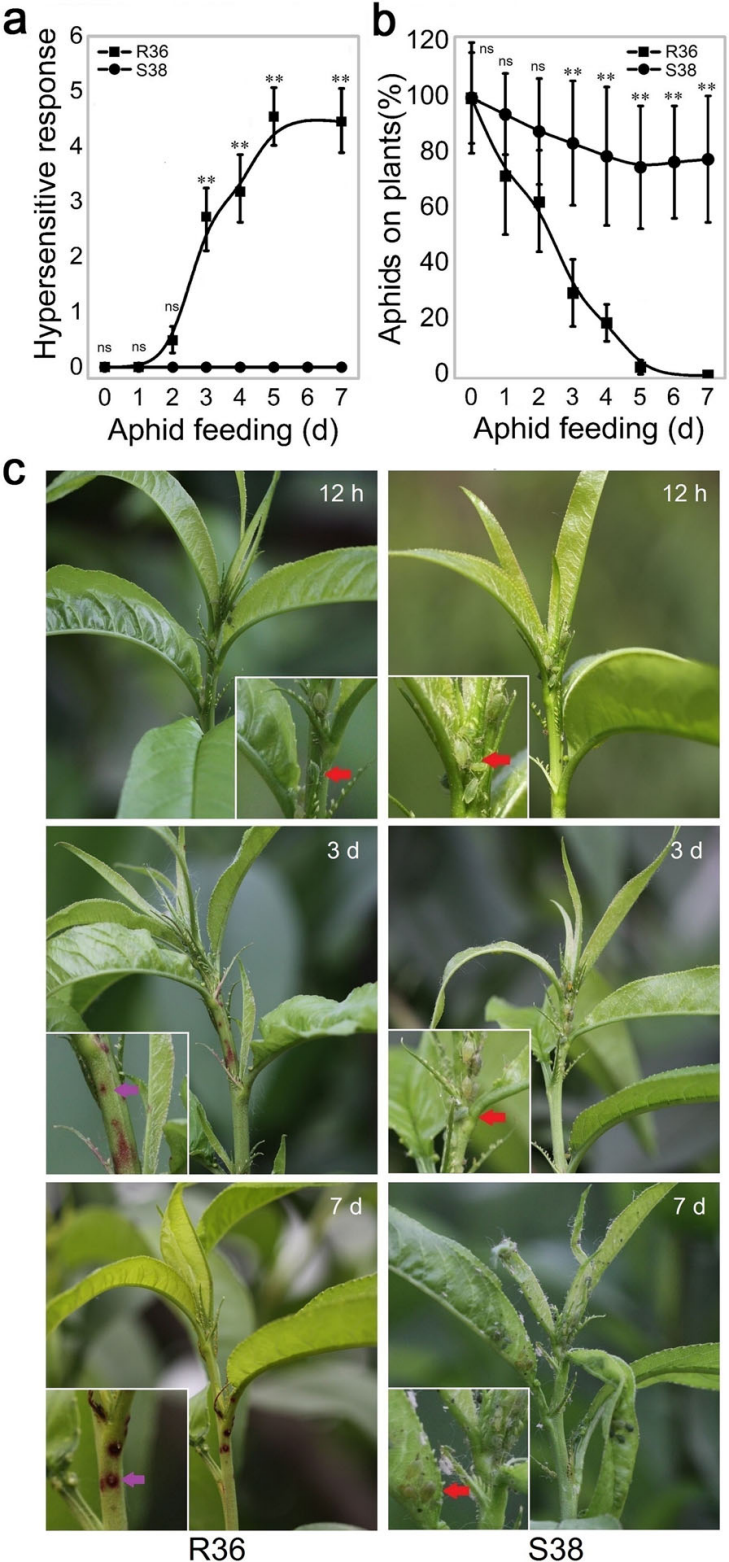
Peach hypersensitive-like reaction development and aphid settlement on R36 and S38. **a** Dynamic changes in the number of hypersensitive-like necrotic speckles after aphid feeding. **b** Percentage of adult aphids remaining on trees. Percentage calculated on 20 shoots, 15 aphids per shoot. **c** Representative pictures for peach hypersensitive-like reactions (purple arrows) and aphid (red arrows) settlement at 12 h, 3d, and 7d. For hypersensitive-like necrotic speckles and adult aphids remaining on trees, the results are the mean ± SE of measurements for at least twenty shoots. Asterisks indicate statistically significant differences compared with ‘S38’ (A) or ‘R36’ (B) at similar days after aphid feeding using Student’s t-test (*P < 0.05, **P < 0.01). ns indicates that there were no significant differences

Young apterous GPA adults were placed on the partially expanded trifoliate leaves of the susceptible individual S38 and the resistant individual R36 from an F1 progeny. The number of hypersensitive-like necrotic reactions on the young stems and the number of adult aphids remaining on the plants were measured 1 week after the aphid infestation. Hypersensitive-like necrotic reactions, an indicator of the presence or absence of *Rm3*-controlled GPA resistance, did not appear in the tender stems of either genotype during the first 2 days and then occurred in R36; however, these reactions were absent in susceptible plants throughout the experiment (Fig. 1a, c). Aphid settlement was similar in resistant individual R36 and susceptible individual S38 over the first 2 days after aphid infestation (DAI, Fig. 1b), as the 12 h picture in Fig. 1c shows. On 2 DAI, the aphids remaining on S38 showed no decrease or a very slow decrease, whereas the aphid number on R36 showed a steady and continuous decrease from 0 DAI to 2 DAI (Fig. 1b). Thus, we hypothesize that the signalling pathways and downstream genes that mediate defence events in the first 3 days of aphid feeding may be crucial to the peach resistance mechanisms. To unravel the molecular mechanisms involved in *Rm3*-type aphid resistance, eight sampling points covering the 3-day time interval were selected for transcriptome analysis.

### RNA sequencing and analysis of shoot-tip transcriptomes

To survey the transcriptomic dynamics that occur in response to aphid feeding, the partially expanded trifoliate leaves of two peach lines, i.e., R36 and S38, were infested with GPAs for 0, 3, 6, 9, 12, 24, 48, or 72 h. mRNA-seq samples were taken at the expected time points. For transcriptome sequencing, RNA was extracted from two biological replicates per sample and converted into cDNA libraries for sequencing. The average number of clean reads for each sample was 47.6 million, of which 88.72% mapped to the *P. persica* v2.0.a1 genome reference sequence [26], which encodes 26,873 annotated genes and 47,089 transcripts (Additional file 1). Read counts per gene were expressed as ‘fragments per kilobase of transcript sequence per millions base pairs sequenced’ (FPKM) mapped reads (Additional file 2). A total of 17,945 genes, approximately two-thirds of the annotated genes in the peach genome, were expressed in at least one sample (FPKM ≥2), and more than half of the expressed genes (13,088) were present in all samples.

### DEG analysis of R36 and S38 after aphid infestation

**Fig. 2:**
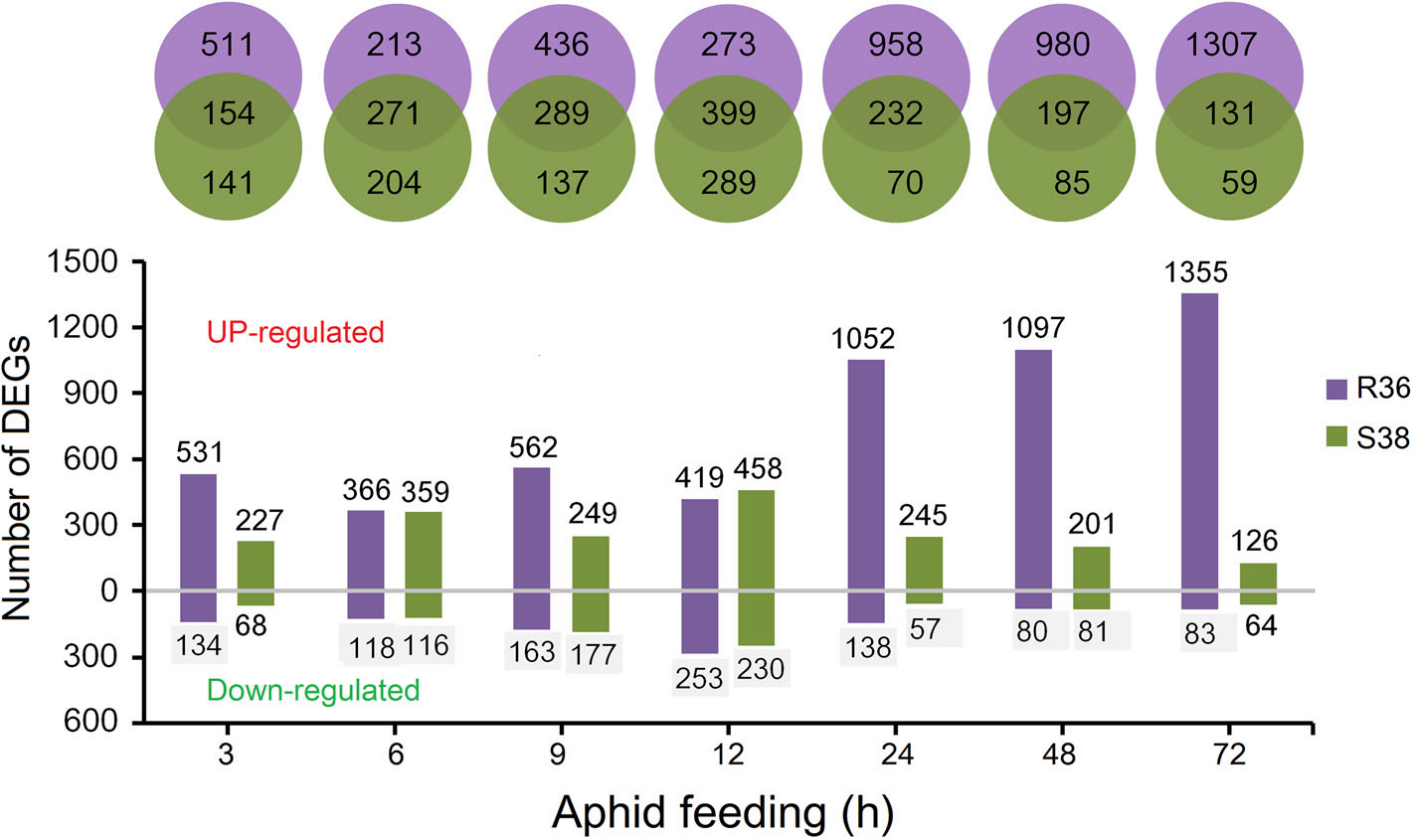
Distribution of differentially expressed genes (DEGs) at different time points after aphid feeding. Venn diagram overlapping regions indicate that the DEGs appeared in both samples represented by the circles. The bar charts represent the distribution of DEGs at different time points. Light purple and light green represent the DEGs in R36 and S38, respectively

To categorize the responses of differentially expressed genes (DEGs) to aphid infestation, the RNA-seq dataset was analysed using the DESeq R package (1.18.0) [27], which identified significant differences in gene expression by pairwise sample comparisons among the sixteen time points. Genes with significant differential expression (*P* < 0.05, false discovery rate [FDR]-adjusted) and a 2-fold or greater change for at least one of the time points were selected, resulting in 3854 genes that were differentially expressed (Additional file 3). We also calculated the number of up- and down-regulated genes according to genotype. After the initiation of aphid feeding, hundreds of transcripts showed altered expression levels for each time point, up-regulated transcripts were much more abundant than down-regulated transcripts in the R36 plants, and the changes in gene expression peaked at 24 to 72 h after infestation (hai) (Fig. 2). Whereas DEG numbers peaked at 24 to 72 hai in R36, the greatest DEG number of induced genes was observed at 12 hai in S38 (Fig. 2). Generally, many more genes were differentially expressed in R36 tissues relative to S38, especially the up-regulated genes from 24 to 72 hai. The number of DEGs at later time points was markedly lower than that in S38 at 12 hai. Over time, the number of DEGs in S38 markedly decreased to 282 at 48 hai and 190 at 72 hai, indicating that the defence response of S38 started to slow down and that the leaves were already colonized at these time points by GPAs (Fig. 1). In contrast, the number of DEGs in R36 did not peak until 72 hai and increased significantly to 1438 at this time, which suggests that the duration of the defence response of R36 was longer and stronger than that of S38, and this peak in the number of DEGs in R36 revealed the significance of these periods. In addition, the analysis identified that more than half of the DEGs of S38 were differentially expressed in both cultivars (Fig. 2).

**Fig. 3:**
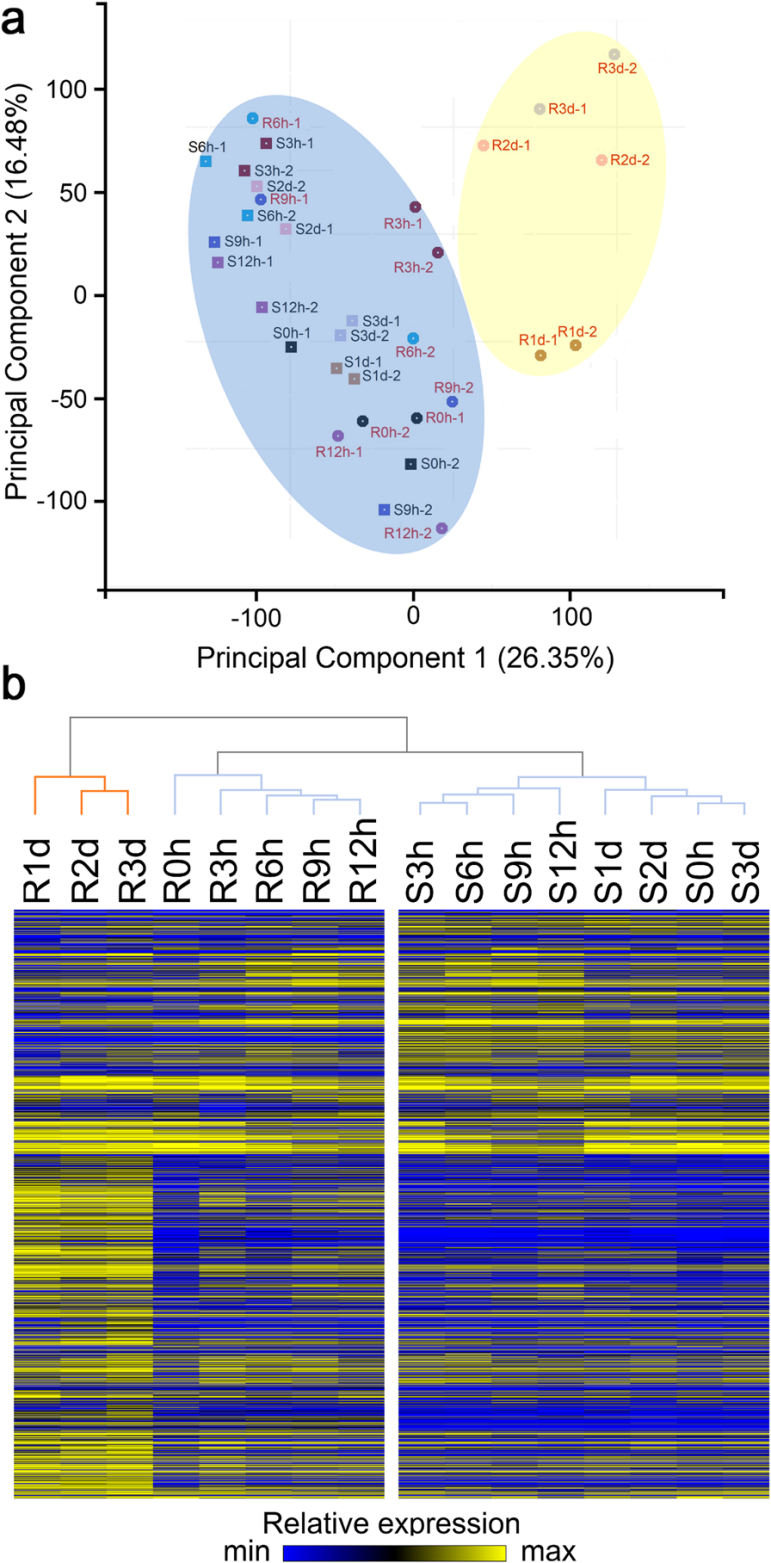
Overview of peach transcriptome responses to aphid feeding. **a** Principal component analysis (PCA) analysis of 3854 genes identified by transcript profiling (RNA-seq) of R36 and S38 infested with aphids for the indicated time periods. Hours of aphid infestation are indicated by the number following the R (R36) or S (S38) cultivar, i.e., R0h indicates 0 h of aphid infestation in R36. **b** Hierarchical clustering of all differentially expressed genes. Distances were calculated using Pearson’s similarity, and agglomeration was performed according to Ward’s minimum variance algorithm. The heat-map diagram shows the relative expression level at the eight time points (0, 3, 6, 9, 12, 24, 48 and 72 h after aphid infection) for two genotypes

To assess transcriptome similarity among samples, principal component analysis (PCA) and hierarchical clustering were performed. Both analyses revealed two discrete groupings: one group consisted of the eight tissues from S38 and the first five time points of R36, and the other group consisted of the 24, 48 and 72 hai time points of R36 (Fig. 3), indicating that after 3 to 12 h of aphid feeding, the gene expression of the two genotypes was similar to that of the non-infested controls, and the greatest changes in gene expression occurred 1 day after the onset of aphid feeding in R36. The overall similarity of the PCA and hierarchical clustering results suggested that the aphid-induced gene expression changes induced changes in the transcriptome at the same or later time points in our experiments.

### Verification of the expression of some DEGs detected during aphid infestation

Quantitative real-time PCR (qPCR) analysis was performed to validate our transcriptome profiling dataset by correlating the qPCR results with the standard RNA-seq data (presented in the Methods). We observed clear positive correlations between the qPCR and RNA-seq data for these two cultivars throughout the whole experimental period after GPA infection (Additional file 4).

### Cluster and gene ontology analysis of DEGs

**Fig. 4:**
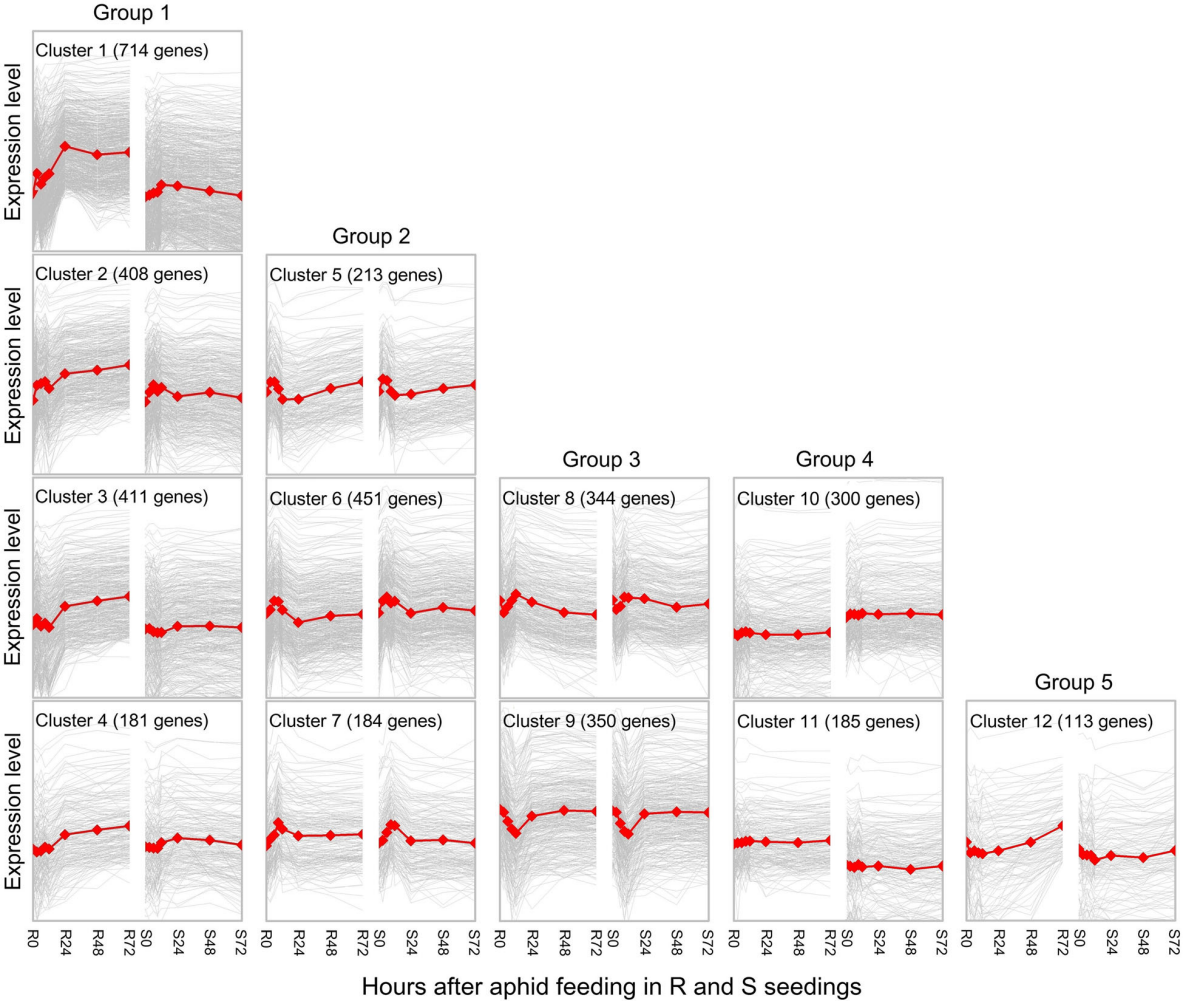
Overview of gene expression clusters calculated by K-means clustering. Pearson correlation was used to identify twelve clusters involving a total of 3854 transcripts with significant expression profile changes for at least one time point after the initiation of aphid feeding. The total number of transcripts in each cluster is indicated, and data for individual genes are shown in light grey. Average expression responses for each cluster are shown in red. R0 and S0 are the control non-infested samples for R36 and S38, respectively. R24, R48, R72, S24, S48, and S72 refer to 24, 48 and 72 h after aphid infestation for R36 and S38. All genes selected for this analysis were significantly differentially expressed by 2-fold or greater (up- or down-regulated), *P* < 0.05 (FDR-adjusted)

The significantly DEGs were input into TM4 software (http://www.tm4.org) for K-means cluster analysis using Pearson correlation distances. Each cluster was represented by the average gene expression level of the genes showing similar response patterns to aphid herbivory. The expression patterns of 3854 DEGs were divided into 12 clusters based on K-means cluster analysis and were classified into five groups manually (Fig. 4). In group I, which was the largest expression profile with 1714 DEGs, the genes were relatively stable both before and after aphid infestation in S38 but were continuously up-regulated in R36 post-infestation. Group I genes were divided into four clusters. In cluster 1 (714 genes), most genes were stable at the all the time points, except for 12 hai, in the S38 line but were continuously up-regulated in the R36 line. Cluster 2 (408 genes) genes were up-regulated from 3 to 12 hai in S36, continuously up-regulated in R36 from 24 to 72 hai, and down-regulated at 24 hai in S36. In cluster 3 (411 genes) and cluster 4 (181 genes), the gene expression profiles did not exhibit any significant differences in S38 but were consistently up-regulated after 24 hai in R36. For the three clusters in group II, the expression profiles of cluster 5 (213 genes), cluster 6 (451 genes), and cluster 7 (184 genes) were similar in both the R36 and S38 lines; gene expression profiles peaked in both lines at 3 hai, 6 hai, or 9 hai in these three clusters. Group III (694 DEGs) gene profiles were also similar in both the R36 and S38 lines. Samples at all time points had similar expression patterns, and gene expression profiles were down-regulated in both lines at 3 hai or 9 hai for cluster 8 or cluster 9. Genes in group IV (485 genes) were stable throughout aphid infestation in both the R36 and S38 lines, with a higher expression level in S38 for cluster 10 (300 genes) and in R36 for cluster 11 (185 genes). In group V (113 genes), the genes were down-regulated from 0 to 24 hai in the R36 line and were subsequently up-regulated from 24 to 72 hai. However, in the S38 line, these genes were stable throughout aphid infestation. Overall, these gene expression differences may account for the contrasting resistance phenotypes in the two individuals from a segregating progeny.

To elucidate the biological processes involved in each gene expression group, overrepresentation analysis was performed using the package ‘GOseq R’. Enrichment of gene ontology (GO) terms was analysed separately for the DEGs, and significant GO terms identified among the DEGs included many terms associated with plant defence responses, stress responses and biotic stimulus responses (Table 1). This analysis provided evidence that calcium signalling and ROS pathways, as well as the production of protein phosphorylation and transcription factors, were up-regulated. Similarly, the observed gene expression patterns indicate that the activation of metabolic processes occurs shortly after aphid feeding, and the most significantly enriched GO terms were metabolic process (GO:0008152), with 736 annotated genes, followed by single-organism process (GO:0044699). Other gene categories related to energy metabolism, including carbohydrate, polysaccharide, and glucan metabolic processes, were also overrepresented among the transcripts that were differentially expressed in response to aphid feeding. Conversely, genes involved in nucleobase-containing compound metabolic processes were overrepresented among the down-regulated genes and underrepresented among the up-regulated genes. Together, these observations indicate that there is a shift in peach from primary metabolism to the production of defensive metabolites in response to GPA feeding.

**Table 1:** Enriched gene ontology (GO) terms identified from the 3854 differentially expressed genes (DEGs) in the R36 and S38 during 24 h after aphid infestation

GO accession	Term type^a^	Term description	*P*_Value	DEG item	DEG total
Group I					
GO:0008152	B	Metabolic process	0.002	736	1257
GO:0044699	B	Single-organism process	0.002	534	1257
GO:0055114	B	Oxidation-reduction process	0.006	177	1257
GO:0006793	B	Phosphorus metabolic process	0.006	152	1257
GO:0006796	B	Phosphate-containing compound metabolic process	0.009	150	1257
GO:0006468	B	Protein phosphorylation	0.006	109	1257
GO:0006950	B	Response to stress	0.006	97	1257
GO:0006952	B	Defence response	0.001	41	1257
GO:0009607	B	Response to biotic stimulus	0.000	33	1257
GO:1901565	B	Organonitrogen compound catabolic process	0.006	19	1257
GO:0006505	B	GPI anchor metabolic process	0.014	17	1257
GO:0046149	B	Pigment catabolic process	0.011	11	1257
GO:0006787	B	Porphyrin-containing compound catabolic process	0.015	11	1257
GO:0033015	B	Tetrapyrrole catabolic process	0.015	11	1257
GO:0051187	B	Cofactor catabolic process	0.015	11	1257
GO:0031012	C	Extracellular matrix	0.030	19	1257
GO:0003824	M	Catalytic activity	0.010	688	1257
GO:0016491	M	Oxidoreductase activity	0.008	180	1257
GO:0004672	M	Protein kinase activity	0.009	113	1257
GO:0048037	M	Cofactor binding	0.023	75	1257
GO:0001071	M	Nucleic acid binding transcription factor activity	0.006	68	1257
GO:0003700	M	Transcription factor activity, sequence-specific DNA binding	0.006	68	1257
GO:0046906	M	Tetrapyrrole binding	0.011	53	1257
GO:0020037	M	Heme binding	0.013	52	1257
GO:0016758	M	Transferase activity, transferring hexosyl groups	0.044	48	1257
GO:0043565	M	Sequence-specific DNA binding	0.027	39	1257
GO:0005509	M	Calcium ion binding	0.023	36	1257
GO:0030246	M	Carbohydrate binding	0.001	32	1257
GO:0001871	M	Pattern binding	0.001	23	1257
GO:0030247	M	Polysaccharide binding	0.001	23	1257
GO:0009378	M	Four-way junction helicase activity	0.045	19	1257
Group II					
GO:0044710	B	Single-organism metabolic process	0.001	149	531
GO:0055114	B	Oxidation-reduction process	0.002	86	531
GO:0005975	B	Carbohydrate metabolic process	0.000	52	531
GO:0044262	B	Cellular carbohydrate metabolic process	0.000	21	531
GO:0005976	B	Polysaccharide metabolic process	0.000	17	531
GO:0006073	B	Cellular glucan metabolic process	0.001	13	531
GO:0005618	C	Cell wall	0.005	13	531
GO:0030312	C	External encapsulating structure	0.008	15	531
GO:0003824	M	Catalytic activity	0.024	304	531
GO:0016491	M	Oxidoreductase activity	0.000	91	531
GO:0016757	M	Transferase activity, transferring glycosyl groups	0.031	32	531
GO:0016758	M	Transferase activity, transferring hexosyl groups	0.002	30	531
GO:0046906	M	Tetrapyrrole binding	0.002	30	531
GO:0020037	M	Heme binding	0.007	28	531
Group III					
GO:1901360	B	Organic cyclic compound metabolic process	0.003	133	463
GO:0046483	B	Heterocycle metabolic process	0.006	128	463
GO:0006725	B	Cellular aromatic compound metabolic process	0.006	128	463
GO:0006139	B	Nucleobase-containing compound metabolic process	0.000	127	463
GO:0009058	B	Biosynthetic process	0.049	126	463
GO:1901576	B	Organic substance biosynthetic process	0.034	123	463
GO:0044249	B	Cellular biosynthetic process	0.040	118	463
GO:0090304	B	Nucleic acid metabolic process	0.026	107	463
GO:1901362	B	Organic cyclic compound biosynthetic process	0.020	87	463
GO:0019438	B	Aromatic compound biosynthetic process	0.024	82	463
GO:0018130	B	Heterocycle biosynthetic process	0.026	82	463
GO:0034654	B	Nucleobase-containing compound biosynthetic process	0.001	81	463
GO:0006260	B	DNA replication	0.000	27	463
GO:0006753	B	Nucleoside phosphate metabolic process	0.038	18	463
GO:0009259	B	Ribonucleotide metabolic process	0.049	15	463
GO:0016747	M	Transferase activity, transferring acyl groups other than amino-acyl groups	0.000	25	463
GO:0016746	M	Transferase activity, transferring acyl groups	0.002	26	463
Group IV					
GO:0043531	M	ADP binding	0.000	24	318

^a^*B* biological process, *M* molecular function, *C* cellular component

### Overview of GPA-induced group I-type transcriptomic changes

**Fig. 5:**
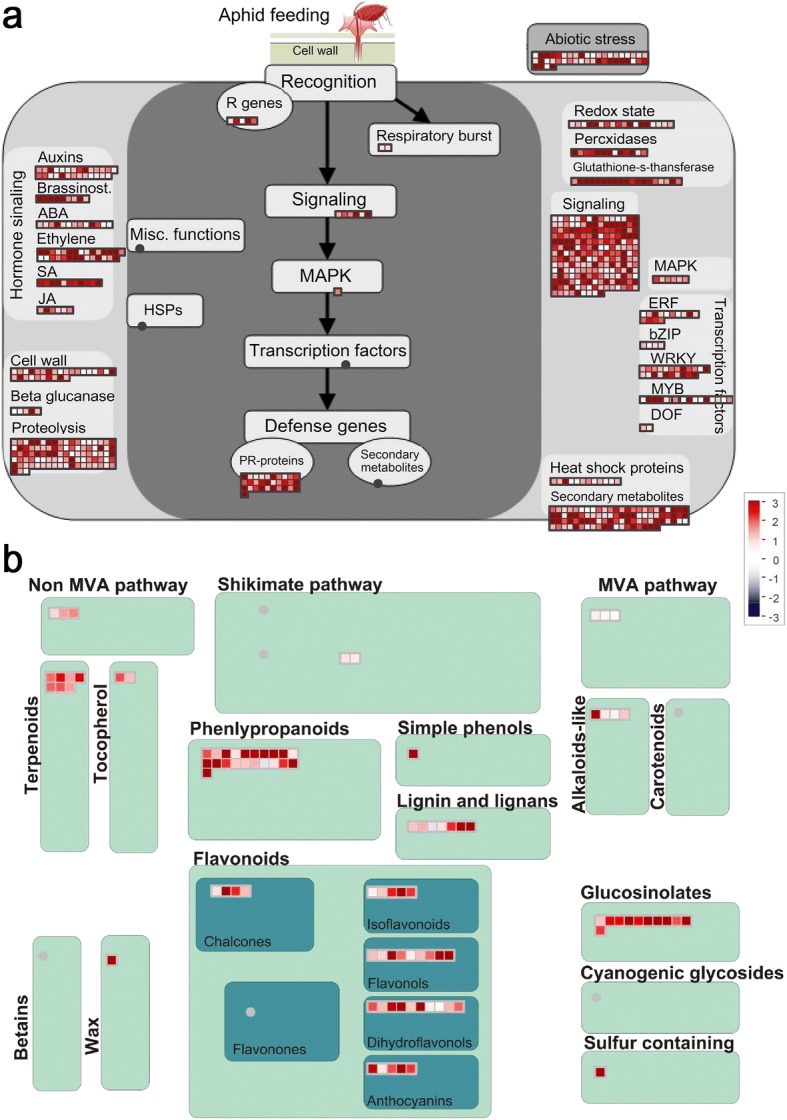
Overview of group I transcripts assigned MapMan biotic stress (**a**) and secondary metabolism (**b**) terms at 24 h after GPA infestation in the resistant line (R36) compared with the susceptible line (S38). The log2-fold changes in the transcript levels were used for the analysis, and the colour scheme on the scale indicates the nature of gene expression; blue means “overexpression in S38”, and red means “overexpression in R36”. Each individual box represents a transcript classified under functional categories (BINs) defined by a MapMan biotic stress pathway

To understand the functional significance of the induced defence responses to GPA infestation, DEGs of group I were imported into the built-in biotic stress and secondary metabolism overview in MapMan software, which classifies the genes putatively involved in mediating biotic stress (pest or pathogen) responses [28]. Two pairwise comparisons between R36 and S38 were used in the functional classification to understand the global changes in response to GPA feeding. The biotic stress response visualization presents substantial alteration in the transcriptomes of both the R36 and S38 genotypes after GPA infestation (Fig. 5a). A total of 681 transcripts belonging to signalling, hormone metabolism, secondary metabolites, cell wall modification, and changes in redox state, peroxidases, glutathione S-transferase (GST), beta glucanases, transcription factors, proteolysis and PR-protein related categories were found to be differentially expressed from 24 to 48 hai. More than 90 secondary metabolism pathway genes were identified in the group I genes, including phenylpropanoid, flavonoid, glucosinolate, lignan and terpenoid pathway genes (Fig. 5b). The annotation details and expression intensities of these differentially expressed transcripts mapped to the biotic stress pathway are presented in Additional file 5.

### Candidate gene analysis of the *rm3* locus in chromosome 1

**Fig. 6:**
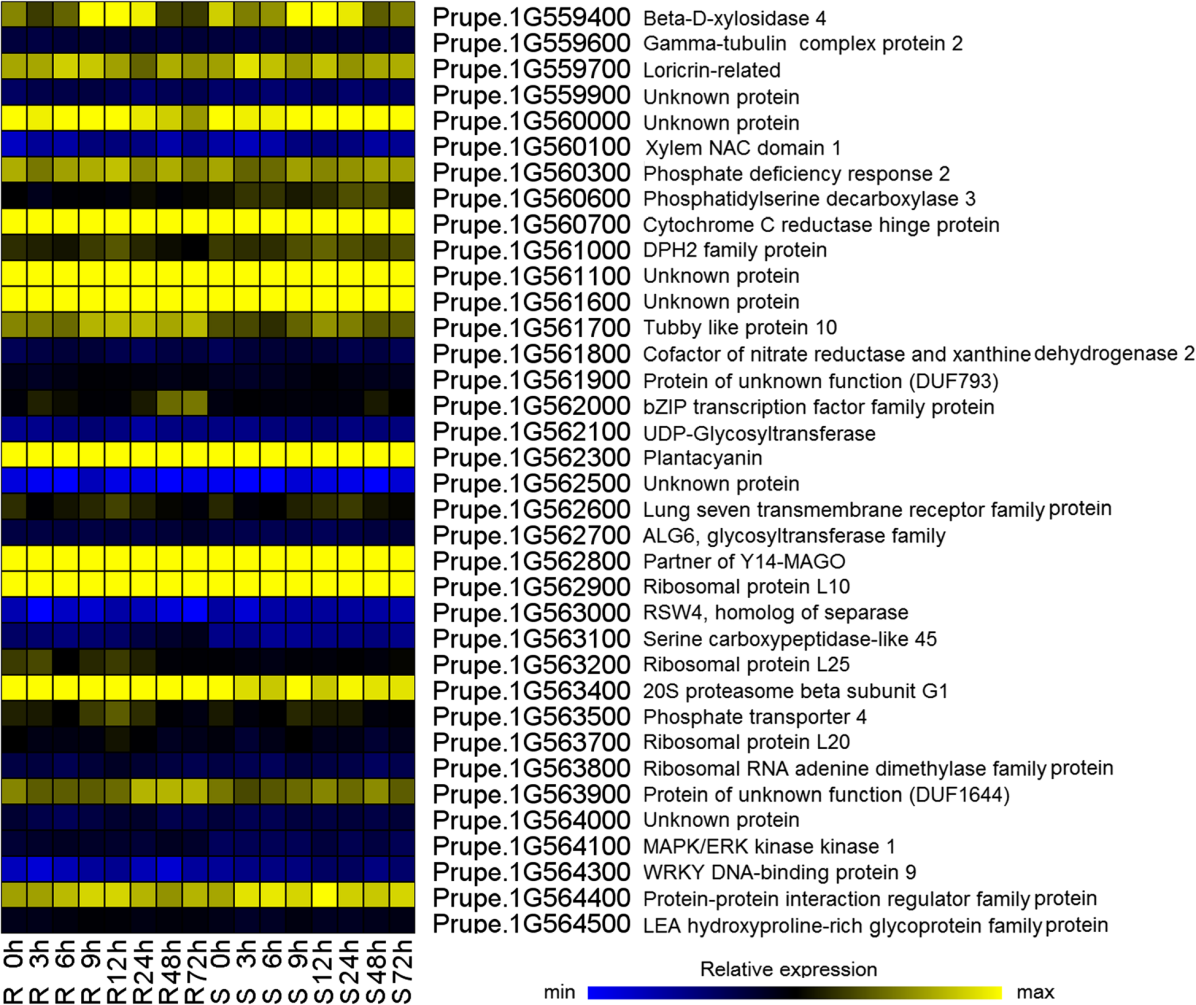
Expression patterns of the expressed genes in the *Rm3* candidate region of chromosome 1 based on the *Prunus persica* v2.0.a1 assembly. R0h and S0 h are the control non-infested samples for R36 and S38, respectively. R3h, R6h, R12h, R24h, R48h, R72h, S3 h, S6 h, S12 h, S24 h, S48 h, and S72 h refer to 3, 6, 9, 12, 12, 24, 48 and 72 h after aphid infestation for R36 and S38

The *Rm3* locus was placed within a 460-kb interval (Pp01:45.66~ 46.12 Mb) on chromosome 1 in our previous study [24]. The published peach genome was sequenced using the aphid-susceptible peach cv. Lovell [29], which does not have *Rm3*-type resistance. Fifty-three genes were annotated in the 460-kb interval containing the *rm3* locus in Lovell, and thirty-six of these genes were expressed (FPKM > 2) in the two genotypes at least one time point (Fig. 6). Based on gene annotation, four genes (Prupe.1G562000, Prupe.1G564100, Prupe.1G562100, and Prupe.1G564300) out of the thirty-six expressed genes may be involved in *Rm3*-type GPA resistance. However, the transcriptome analysis showed that only eleven of these genes had differential expression patterns in the leaves of R36 and S38 after GPA feeding (Additional file 6). The Prupe.1G564300 gene (WRKY DNA-binding protein 9) was the only identified candidate gene based on gene annotation and gene expression analysis.

## Discussion

The genetics of peach resistance to aphids have been studied in detail. However, genomic research of peach-GPA interactions lags far behind. Although *M. persicae* is a pest of 40 different host plant families (including *Brassicaceae*, *Solanaceae*, *Rosaceae*, and *Fabaceae*), biotic stress responses following infestation by this generalist phloem-feeding insect have been established only for the model plants *Arabidopsis* and *Solanum stoloniferum* [25]. The main goal of this study was to understand the molecular mechanism underlying the dominant resistance to *M. persicae* linked to *Rm3* [21, 24]. In the present study, two lines from an F1 progeny, R36 and S38, segregating for *Rm3* resistance traits in the bottom of chromosome 1 were compared using high-throughput sequencing technology. This approach allowed us to compare the changes in gene expression under *M. persicae* infection in the resistant and susceptible genotypes and to identify genes that were specifically induced in the resistant cultivar in response to infection.

### Aphid resistance is activated in R36

The different transcriptomes of two peach genotypes were evaluated both before and after GPA infestation (Fig. 1) using high-throughput Illumina sequencing technology. These peach lines differ in their resistance to *M. persicae* (Sülzer) controlled by the *Rm3* locus at the bottom end of chromosome 1 [24] but are genetically related via the use of GPA-resistant lines from the parent wild peach material “Fen Shouxing”. The aphid-resistant line R36 showed stronger antixenosis resistance than S38, which was fully susceptible to GPAs (Fig. 1). Hypersensitive-like necrotic reactions in the form of reddish or yellowish spots and rejected aphid settlement were observed in R36 but not in S38, while in S38, the apical stems and leaves were fully colonized by GPAs, and the considerable GPA invasion ultimately led to leaf curl. These results suggest that the *Rm3* locus was sufficient to confer a monogenic resistance to aphids that was associated with the HR reaction and GPA escape behaviour, which is similar to the dominant genetic resistance established for both “Weeping Flower Peach” [30] and “Rubira®” [3].

Although this common defence-like response indicates that both lines can perceive *M. persicae* (Sülzer), the susceptibility of S38 clearly shows that the response of S38 is not sufficient to control this aphid. A comparison of the DEGs between R36 and S38 provided clues to the mechanisms that may explain the *Rm3*-type GPA resistance. To unravel the molecular mechanisms involved in this resistance, the transcriptomes of both lines were compared between non-infested plants and plants sampled from 3 to 72 hai, a key infection time interval in which the reddish or yellowish hypersensitive-like necrotic spots appeared on the apices at the puncture points and more than half of aphids left the resistant plants (Fig. 1). Overall, 3854 DEGs were identified. These DEGs are considered to encompass the most relevant genes for GPA defence because they include not only DEGs between the aphid-resistant and aphid-susceptible lines at eight key time points after aphid feeding but also DEGs between infested and non-infested plants at the same time point within R36 or S38, which account for aphid defence responses (in R36) that may not be captured by a direct comparison between R36 or S38. There were markedly more up-regulated DEGs than down-regulated DEGs in the comparisons between the aphid-exposed samples and the controls in both genotypes. Additionally, the number of up-regulated DEGs in the R36 genotype was higher than that in the S38 genotype (Fig. 4). Taken together, these results suggest that aphid infestation resulted in more activated than repressed genes in both the R36 and S38 genotypes, and the genes involved in defensive reactions to aphid infestation were activated at higher levels in the R38 genotype than in the S38 genotype.

### The *Rm3* locus is associated with the induced expression of defence and signalling genes

Among the genes that were induced more strongly in the resistant peach genotype than the susceptible peach genotype, we detected a significant enrichment in functional classes directly related to biotic stress resistance. These included genes associated with crucial biological processes, such as oxidation-reduction process, protein phosphorylation, stress response, defence response, biotic stimulus response and organonitrogen compound catabolic process terms (Table 1). The most significant up-regulation was observed for important protein families including WRKY transcription factors, ERF transcription factors, MYB transcription factors, PR proteins, and several secondary metabolism biosynthetic enzymes that play important roles in GPA resistance ((Fig. 5). Among these proteins, WRKYs are well known for regulating plant abiotic and biotic stress tolerance. These WRKY TFs specifically bind the cis-elements (TTGACC/T; W-box) in the promoter regions of their target genes, and the role of TFs in plant defences against pathogens and herbivores is well known. In rice, silencing *OsWRKY45* reduced the feeding, oviposition preference, and survival rate of *N. lugens* [31]. *Arabidopsis WRKY33* plays an important role in resistance to necrotrophic pathogens, and transgenic plants constitutively expressing the *WRKY33* gene are more resistant than wild-type plants to necrotrophic pathogens [32]. We also observed the up-regulation of this gene family in peach in response to *M. persicae* infection, and the observed changes were stronger in the resistant line than in the susceptible line (Fig. 5). The induction of WRKY TF expression was more pronounced in resistant genotypes under *M. persicae* infection than in susceptible genotypes under *M. persicae* infection. Moreover, under stress conditions, the WRKY TF expression level was significantly higher in the resistant line than in the susceptible line, whereas under control conditions, there was no significant difference between the resistant and susceptible genotypes. In our case, the defence strategy conferred by *Rm2* led to induced defences, which are efficient in terms of plant energetics and are economic and effective ways to protect plants from damage, allowing plants to quickly respond to aggressive and fast-spreading pathogens. Special attention should be paid to these genes in future searches for resistance gene candidates.

### Secondary metabolism pathways may be crucial for GPA resistance

Plants have evolved sophisticated defence mechanisms to combat herbivore invasion; for example, plants produce specialized morphological structures and secondary metabolites and proteins that have toxic, repellent, and/or antinutritional effects on herbivores [33]. Energy and nutrition are redistributed from growth and development to the synthesis of secondary metabolites that play a defensive role, resulting in protection from the attacker [34]. Cluster analyses showed that more than half of the DEGs (infested vs. non-infested) in both genotypes were differentially regulated between the two cultivars in response to *M. persicae* Sülzer infestation (Fig. 2). Genes in the phenylpropanoid, flavonoid, glucosinolate, lignan, alkaloid and terpenoid pathways were the most significantly induced and enriched at both time points, and more than 90 genes in secondary metabolism pathways were identified in the group I genes (Fig. 4). Phenylpropanoid metabolism transforms phenylalanine (Phe) into the majority of the phenolic compounds found in nature, including lignins, sinapate esters, stilbenoids and flavonoids, which play important roles in plants, especially as pigments and defence compounds [35]. Phenolic compounds such as flavonoids, lignans, stilbenes and tannins comprise a major class of inducible defence compounds in many woody species [33]. In peach, metabolome analyses have revealed that the accumulation of phenolic compounds (dicaffeoylquinic acids) in response to *M. persicae* infestation is correlated with *Rm2*-type GPA resistance [36]. In this study, GPA infestation resulted in the differential regulation of seven lignan pathway-related transcripts and thirty-three flavonoid pathway-related transcripts putatively associated with *M. persicae* infestation responses (Fig. 5b, Additional file 5). Alkaloids belong to a structurally diverse group of nitrogen-containing basic natural products with many targets and biological activities, such as interfering with nervous systems, the disruption of DNA synthesis and repair, and the inhibition of protein synthesis. The pea aphid (*Acyrthosiphon pisum*) is strongly deterred by indolizidine and quinolizidine alkaloids [37]. Interestingly, studies have shown that aphids may tolerate low concentrations of alkaloids, as the accumulation of these alkaloids provides a clear defensive benefit [38]. Glucosinolates are a group of anionic thioglucosides present in *Brassicaceae* [39] and do not seem to be toxic in the absence of their hydrolysing enzyme myrosinase. Upon plant tissue damage, glucosinolates co-occur with myrosinase and are rapidly hydrolysed to toxic isothiocyanates (mustard oils) [40]. After herbivore feeding, plants release high levels of mustard oil, which is a very effective defence against some generalist herbivores [41]. Aphids avoid activating this response by causing minimal damage to cells and thus consume and exude mostly intact glucosinolates with little negative effects [42]. Two aphid species, *Brevicoryne brassicae* and *Lipaphis erysimi*, sequester intact host plant glucosinolates and convert these glucosinolates to toxic mustard oils by using their own myrosinase [43, 44]; thus, glucosinolates have negligible or even beneficial effects on aphids. Terpenes are major components of plant volatile organic compounds (VOCs) and often play important roles in the plant defence by attracting enemies or predators of herbivores and repelling herbivorous insect feeding. The (E)-β-farnesene (EBF), an alarm pheromone towards aphids, is emitted by the *M. persicae*-infested host plant [44, 45]. Based on the above analysis, although there are many DEGs in the metabolic pathways of terpenoids and glucosinolates, we believe that the secondary metabolites (including dicaffeoylquinic acids [36]) from the phenylpropanoid/flavonoid metabolic pathway are the most important defence responses for peach trees against GPAs.

### Gene expression analysis of the *rm3* locus

In the past two decades, a number of loci conferring resistance to phloem-feeding insects have been identified and mapped. Including *Rm1*, *Rm2* and *Rm3*, many mapped resistance loci show a strong homology or tight linkage with the NBS-LRR resistance protein gene family [23, 46–48], and five cloned R loci to date all control hemipteran insect resistance, suggesting that the *Rm*-type gene is also a member of the NBS-LRR family [49]. NBS-LRR genes were up-regulated at high levels in host plants in response to biotic stresses [50–53]. The finely mapped genomic interval of *Rm3* partly overlaps with the (~ 1.15 Mb) region of *Rm2* (Pp01:45.08 ~ 46.23 Mb) [22, 24] and with the candidate genomic region (2.88 Mb) of *Rm1* (Pp01:43.62 ~ 46.50 Mb) [23]. The mapped loci of the three type *Rm* genes are at the bottom of chromosome 1, which is flanked by NBS-LRR resistance gene analogues [54]. However, in this study, there was no differentially expressed R gene from the finely mapped interval of *Rm3*-type genes (Fig. 6). As transcriptome data provide absolute rather than relative expression levels, the transcriptome can be used to identify the unexpressed genes in R36 and S38. Among the fifty-three genes annotated in the 460-kb interval containing the *rm3* locus, thirty-six genes were expressed (Fig. 6, Additional file 6). These genes, especially the 7 genes with different expression levels in R36 and S38 after aphid infection, may be candidate genes for *Rm3* (Fig. 6). Remarkably, only 1 out of the 7 genes was defence related; this gene is a WRKY DNA-binding protein gene (Prupe.1G564300). It is worth investigating the function of the two unknown genes because they may present a new explanation for the molecular mechanism of GPA resistance in peach. Considering the wild peach origin of the *Rm3* gene, it is also plausible that this study may not be able to capture the regulatory gene in the *rm3* locus because the genome of “lovell” does not possess antixenosis resistance to GPAs. We cloned a novel R gene from aphid-resistant peach lines derived from ‘Shouxing Tao’, and this gene co-segregated with *Rm3*-type aphid resistance (unpublished data). The protein encoded by this novel R gene could be responsible for the large number of DEGs, i.e., the disease resistance (R) genes, by controlling the specific ability to sense viral pathogen invasions and subsequently trigger a series of downstream immune responses [55]. Functional analysis of the novel R gene is currently underway to understand the mechanism of *Rm3* resistance.

## Conclusions

In the present study, a comparison between the expression profiles of the resistant peach line R36 and the susceptible peach line S38 during the early stage of *M. persicae* Sülzer-*P. persica* interaction revealed that the regulation of defence responses against *M. persicae* Sülzer was clearly different between the two genotypes. Genes mainly involving biotic stimulus and stress, the ROS scavenging system, TFs, PR proteins, and secondary metabolites including phenylpropanoids/flavonoids were more strongly up-regulated in the resistant genotype than in the susceptible genotype. Although it is not clear whether these genotype-specific differences in the transcript abundances of defence-related genes contribute to *M. persicae* Sülzer resistance in *P. persica*, these new discoveries support future experiments that may uncover the mechanisms responsible for *Rm3*-dependent GPA resistance in *P. persica*.

## Methods

### Plant materials, aphid infestation, and sampling

Two individuals from an F1 peach progeny were used in this study. The progeny was derived from a cross between ‘Chun Mei’, an aphid-susceptible peach (*rm3*/*rm3*), and ‘01–29-23’, an aphid-resistant peach (*Rm3*/*rm3*), which had been segregated for *Rm3* resistance traits. ‘01–29-23’ was derived from ‘Shouxing Tao’ (*P. persica* var. densa Makino), and genetic analysis indicated that the GPA resistance of ‘Shouxing Tao’ is controlled by a single dominant gene in linkage group 1 linked to the peach flesh colour (white or yellow) [21, 56]. This progeny consisted of 31 seedlings. Two lines, ‘07–15-36’ (aphid-resistant, *Rm3*/*rm3*) and 07–15-38 (aphid-susceptible, *rm3*/*rm3*), referred to as R36 and S38, respectively, were used for aphid infestation and RNA-seq library construction. Trees were planted in a field at the ZFRI Experimental Station at Zhengzhou (Henan, China). The trees were planted in a 4 × 2.0 m arrangement with drip irrigation and fertilizer applications as required. The test aphids were derived from a clonal culture of *M. persicae* originating from a single female collected on a peach tree grown in an orchard in the Zhengzhou Fruit Research Institute, China. This clone has been continuously reared on susceptible seedlings of peach plants (variety CN14) under parthenogenesis-inducing conditions of 20 °C and 16-h-light/8-h-dark photoperiod in a growth chamber [18]. For aphid infestation treatment, fifteen adult aphids were placed on the partially expanded trifoliate leaves of the susceptible and resistant individuals. Before sampling, the remaining aphids were carefully removed from the leaves. Five shoot tips were sampled at time 0 (no aphid infestation), 3, 6, 9, 12, 24, 48, and 72 hai. Collected materials were immediately frozen in liquid nitrogen and stored at − 80 °C until use.

### Aphid assays

To assess the plant-colonizing ability of GPAs, fifteen apterous adults were deposited on the terminal shoots of the resistant and susceptible plants. The number of adults remaining alive on the plants over a period of 7 days was recorded. During this period, the number of hypersensitive-like necrotic reactions (a type of reddish or yellowish spots) was also recorded. The parameters were collected from 20 shoots for each plant.

### RNA-seq library construction and high-throughput sequencing

Total RNA from the peach shoots was extracted using the RNAprep Pure Plant Kit with on-column DNase digestion (Tiangen Biotech Co., Ltd., Beijing, China) according to the manufacturer’s instructions. RNA purity and concentration were measured using a NanoPhotometer spectrophotometer (IMPLEN, CA, USA), and the RNA integrity was confirmed using the RNA Nano 6000 Assay Kit from the Bioanalyzer 2100 system (Agilent Technologies, CA, USA). A total of 32 RNA-seq libraries (two biological replicates at eight time points for both the aphid-resistant and susceptible peach lines) were constructed as described previously [57] using the NEBNext Ultra Directional RNA Library Prep Kit for Illumina (New England Biolabs, Ipswich, MA) following the manufacturer’s recommendations, and index codes were added so sequences could be attributed samples. The clustering of the index-coded samples was performed on a cBot Cluster Generation System using the TruSeq PE Cluster Kit v3-cBot-HS (Illumina) according to the manufacturer’s instructions. After cluster generation, the library preparations were sequenced on an Illumina HiSeq 4000 platform, and 150 bp paired-end reads were generated. The whole library construction and Illumina sequencing step was performed at Novogene Bioinformatics Technology Co., Ltd., Beijing, China (www.novogene.cn).

### RNA-seq data analysis

Clean data (clean reads) were obtained by removing reads containing adapters, reads containing ploy-N and low-quality reads from raw data with in-house Perl scripts. At the same time, the Q20, Q30 and GC content values of the clean data were calculated. All downstream analyses were based on high-quality clean data. Reference genome and gene model annotation files were downloaded from GDR (URL: https://www.rosaceae.org/species/prunus_persica/genome_v2.0.a1) [29]. The reference genome index was built using Bowtie v2.2.3 [58], and paired-end clean reads were aligned to the reference genome using TopHat v2.0.12 [59]. Relative transcript abundance was calculated based on fragments per kilobase of transcript sequence per millions base pairs (FPKM) for biological replicates. HTSeq v0.6.1 was used to count the read numbers mapped to each gene [60], and the differential expression of genes was determined with Cuffdiff (v.2.1.1), with bias correction applied to all samples.

### RNA extraction, complementary DNA synthesis and quantitative real-time PCR

To validate the RNA-seq data, the expression of three biological replicates of fourteen genes (approximately 0.5% of all differentially regulated genes) was investigated by qPCR. RNA was extracted using the RNAprep Pure Plant Kit (TIANGEN, Beijing, China) according to the manufacturer’s instructions. RNA quality, purity, and concentration were assessed using a Nano-Drop 2000 spectrophotometer. First-strand complementary DNA was synthesized using the FastQuant RT Kit (TIANGEN, Beijing, China) according to the manufacturer’s instructions. All gene-specific primers were designed according to EST sequences using Primer Premier 5.0 (Additional file 7). Amplification reactions were run in a 96 well plate on a LightCycler 480 (Roche) with a LightCycler 480 SYBR Green I Master Kit (Roche Molecular Biochemicals), as previously described [59] under the following conditions: 95 °C for 30 s, followed by 45 cycles of denaturation at 95 °C for 10 s, annealing at 60 °C for 10 s and extension at 72 °C for 15 s. Amplifications were normalized to *actin*, and each reaction was performed with three biological replicates for each sample. The relative expression levels were determined as described previously [61].

### GO enrichment analysis and MapMan analysis

To evaluate functional activities differentially represented in aphid-susceptible or aphid-resistant lines, we mapped the DEGs to known biological ontologies based on the GO project (http://www.geneontology.org/). Enrichment analysis was performed on these sets using the R package ‘GOseq’, which accounts for biases because of the over-detection of long and highly expressed transcripts [62]. Gene sets with ≤10 genes were excluded from the analysis. We used the REVIGO web page [63] to summarize and remove redundant GO terms from the results. Only GO terms with a FDR < 0.05 were used. REVIGO plots were obtained for two GO categories: biological processes and molecular functions. The GO project describes a biological process as a recognized series of events or molecular functions with a defined beginning and end and describes molecular functions as activities that occur at the molecular level, such as catalytic or binding activities. The DEGs were clustered based on their expression patterns by the Genesis K-means method [28].

MapMan [28] was used for data visualization. False colour imaging was performed on the log_2_-transformed RNA-seq data. RNA-seq data were the average values of all replicates for each time point.

## Additional files

Additional file 1: Number of reads sequenced and mapped in each sample. (XLSX 11 kb)

Additional file 2: Gene expression in peach leaves after aphid infestation. (XLSX 11389 kb)

Additional file 3: RNA-seq data for sixteen aphid feeding time points after analysis by DESeq R. (XLSX 17265 kb)

Additional file 4: Comparison of RNA-seq and qPCR gene expression data. (DOCX 444 kb)

Additional file 5: Enriched MapMan category terms for the significantly differentially expressed genes of group I altered by aphid feeding. (XLS 2705 kb)

Additional file 6: Gene annotation and expression of the fine mapped *Rm3* locus. (XLSX 20 kb)

Additional file 7: Primers used for quantitative qPCR analysis. (XLSX 10 kb)

## Abbreviations

DEGs: Differentially expressed genes
ETI: Effector-triggered immunity
GPA: Green peach aphid
HR: Hypersensitive response
MAMPs: Microbe-associated molecular patterns
PPV: Plum pox potyvirus
PR: Pathogenesis-related
PRRs: Plasma membrane-localized pattern recognition receptors
PTI: Pattern-triggered immunity
ROS: Reactive oxygen species
