# Supplementary material for: Dynamic transcriptomes of resistant and susceptible peach lines after infestation by green peach aphids (Myzus persicae Sülzer) reveal defence responses controlled by the Rm3 locus
Source: BMC Genomics. 2018 Nov 28;19:846. doi: 10.1186/s12864-018-5215-7 (PMC6264056; doi:10.1186/s12864-018-5215-7)
Supplement: Supplementary file 4 — Comparison of RNA-seq and qPCR gene expression data. (DOCX 444 kb) [file 12864_2018_5215_MOESM4_ESM.docx]

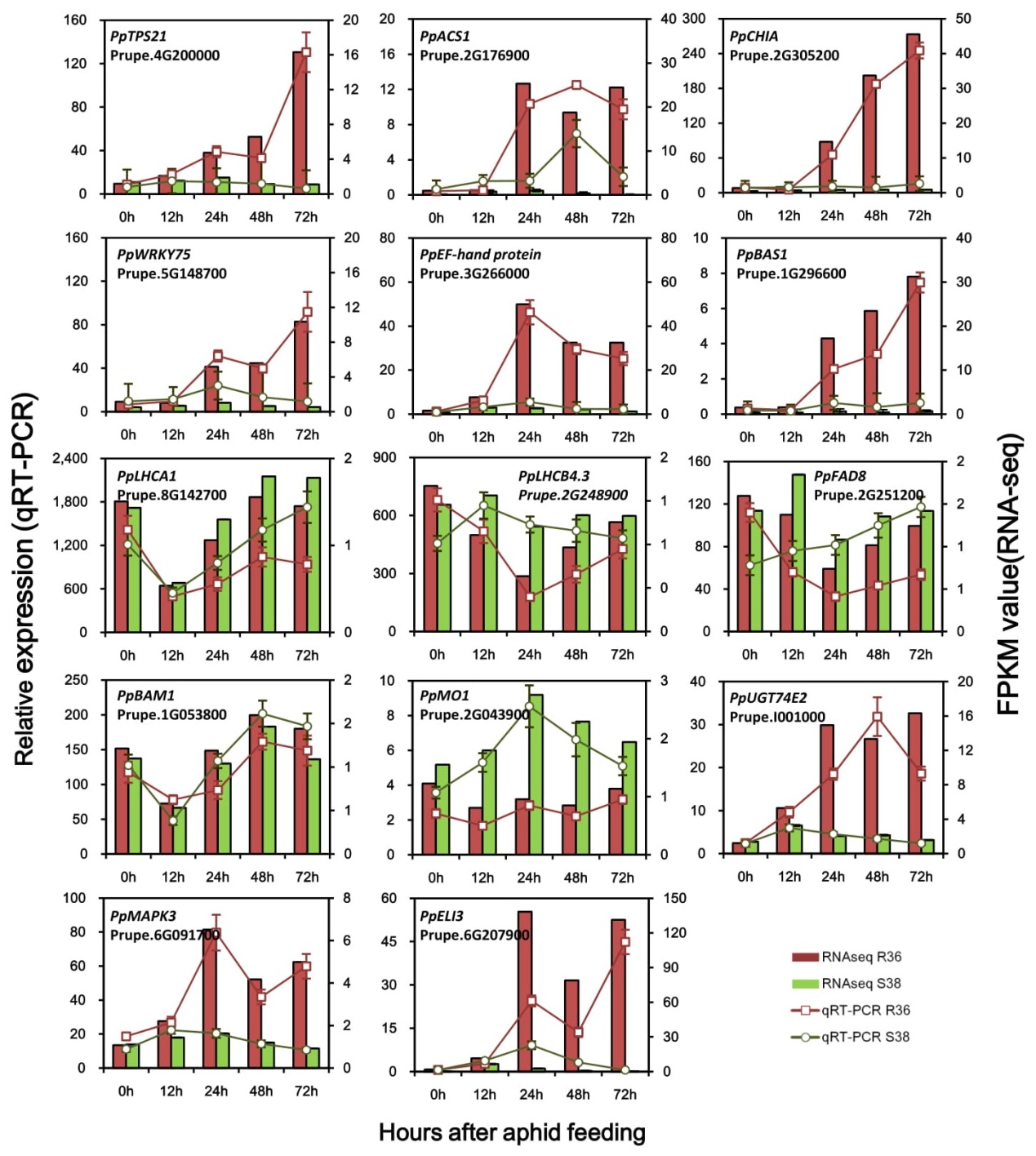


Additional file 4. Comparison of RNA-seq and qPCR gene expression data. The fourteen genes are assigned to the resistance signaling pathway, including photosynthesis, kinases and transcription factors and other uncharacterized genes. Columns represent expression by RNA-seq in FPKM values (left y-axis), while lines represent expression determined by qRT-PCR (right y-axis). The x-axis in each chart represents the five time points (0, 12, 24, 48 and 72h). For qRT-PCR, normalization was made to expression of the actin gene, and values are means of three technical replicates. For qRT-PCR, normalization was made to expression of the *actin* gene, and values are means of three biological replicates.
